# Self-Avoiding Random Walks as a Model to Study Athermal Linear Polymers under Extreme Plate Confinement

**DOI:** 10.3390/polym12040799

**Published:** 2020-04-03

**Authors:** Oscar Parreño, Pablo Miguel Ramos, Nikos Ch. Karayiannis, Manuel Laso

**Affiliations:** Institute for Optoelectronic Systems and Microtechnology (ISOM) and Escuela Técnica Superior de Ingenieros Industriales (ETSII), Universidad Politecnica de Madrid (UPM), José Gutierrez Abascal 2, 28006 Madrid, Spain; o.parreno@alumnos.upm.es (O.P.); pm.ramos@alumnos.upm.es (P.M.R.); mlaso@etsii.upm.es (M.L.)

**Keywords:** confinement, crystallization, entropy, hard sphere, polymer, random walk, Monte Carlo, phase transition, lattice model, cubic crystal system, direct enumeration

## Abstract

Monte Carlo (MC) simulations, built around chain-connectivity-altering moves and a wall-displacement algorithm, allow us to simulate freely-jointed chains of tangent hard spheres of uniform size under extreme confinement. The latter is realized through the presence of two impenetrable, flat, and parallel plates. Extreme conditions correspond to the case where the distance between the plates approaches the monomer size. An analysis of the local structure, based on the characteristic crystallographic element (CCE) norm, detects crystal nucleation and growth at packing densities well below the ones observed in bulk analogs. In a second step, we map the confined polymer chains into self-avoiding random walks (SAWs) on restricted lattices. We study all realizations of the cubic crystal system: simple, body centered, and face centered cubic crystals. For a given chain size (SAW length), lattice type, origin of SAW, and level of confinement, we enumerate all possible SAWs (equivalently all chain conformations) and calculate the size distribution. Results for intermediate SAW lengths are used to predict the behavior of long, fully entangled chains through growth formulas. The SAW analysis will allow us to determine the corresponding configurational entropy, as it is the driving force for the observed phase transition and the determining factor for the thermodynamic stability of the corresponding crystal morphologies.

## 1. Introduction

Polymer based thin films have been used extensively for several decades in a varied range of applications from optical coatings and energy storage to smart appliances, semiconductors and pharmaceutics [1,2,3,4,5,6,7,8,9,10,11,12,13,14,15,16,17,18,19]. Further inclusion of nanoparticles or adhesion to complex interfaces allows efficient control, tuning, and magnification of the already exceptional base macromolecular properties. To achieve superior characteristics, it is paramount to understand the complex structural and dynamic behavior of macromolecules, ideally at the level of atoms and molecules, under confinement and to relate them with macroscopic properties of the end material.

Especially relevant for numerous applications is the phase behavior, as macromolecular crystals exhibit distinctly different characteristics compared to polymer glasses. While phase transition, as observed in complex atomic systems, is extensively studied it is still far from being fully understood. The early work of Alder and Wainwright, based on collision-driven molecular dynamics (MD), demonstrated that monomeric hard spheres crystallize at high concentrations [20]. It is now established that given enough observation time, hard spheres or corresponding colloidal entities show spontaneous crystallization under a variety of conditions (microgravity, impurities, size polydispersity etc) once a critical range of packing densities is reached [21,22,23,24,25,26,27,28,29,30,31,32,33,34,35,36,37,38,39,40,41,42]. Given the athermal nature of such systems, entropy is the driving force of crystal nucleation and growth and dictates the resulting, thermodynamically stable, ordered morphologies. Recently, it has been demonstrated that dense packing of chains of hard spheres also crystallize [43,44,45,46]. It is possible to effectively control the phase behavior and/or the state of jamming [47,48,49,50] for polymers by properly tuning the bond gaps (or bond tangency) between successive monomers along the chain [51,52] or the bending angles that dictate chain flexibility [53,54]. Similar control can be achieved by applying spatial confinement; in the most trivial case this can be realized through the presence of flat, impenetrable, and parallel walls in at least one dimension.

In the past, we used a Monte Carlo (MC) scheme [55], built around chain-connectivity-altering MC moves [56,57,58,59], to generate and equilibrate freely-jointed chains of hard spheres of uniform size in the bulk. This allowed us to systematically study the effect of packing density, chain length and bond tangency/gaps on the local and global structure of athermal macromolecules [48,60,61], on the primitive path network of entanglements [49,62] and on the ability of chains to crystallize [43,44,45,46,52]. Recently, based on the original scheme of Ref. [55], we introduced a more general method including a wall-displacing algorithm which allows the simulation of athermal polymer packings under confinement [63]. This more general method has allowed us to simulate systems of very long chains under extreme confinement and at very high packing density and at high cell shape anisotropy. For the linear chains considered here the number of monomers is, *N*_mon_ = *N* + 1, where *N* is the number of bonds. Confining agents are flat, parallel and impenetrable surfaces (walls). Packing density, *ϕ*, is defined as the volume occupied by all chain monomers divided by the volume of the simulation cell. The number of confined dimensions, *d*_conf_, ranges from zero (unconstraint, bulk case) to three (fully confined). Extreme confinement is reached when the distance between the walls, *d*_wall_, in at least one dimension approaches the size of sphere monomers, *σ*. Cell shape anisotropy is quantified through the cell aspect ratio, *ζ*, which is the ratio of longest length(s) divided by the shortest one(s). In all cases cell shape corresponds to an orthogonal parallelepiped.

Through the proposed MC scheme we equilibrate dense athermal chain packings under extreme confinement that correspond to quasi-1D (tube-like) and quasi-2D (plate-like) polymer thin films [63]. In both cases as a critical combination of concentration and confinement is reached polymers transit to ordered morphologies characterized by structural defects. For plate-like packings this ordered state is a blend of hexagonal close packed (HCP) and face centered cubic (FCC) domains. Given that the system is athermal, any phase transition is driven by a change (increase) in the total entropy. Accordingly, to predict the phase transitions and to identify the thermodynamically stable phase, a first step is to calculate the configurational entropy of chains. Towards this, we map the corresponding atomistic chains onto self-avoiding random walks (SAWs) grown step-by-step on regular lattices subject to specific spatial restrictions. During the growth, the next position to lattice has to be adjacent to the current one. Self-avoidance condition dictates that no lattice point can be visited twice. In addition, the imposed spatial restrictions on SAW lattices mimic the ones encountered at the atomistic level. In a previous paper we enumerated the total number of SAWs for two different lattices SC (simple cubic) and FCC as a function of the system geometry and the number of chain bonds (or equivalently SAW steps) for quasi-1D, tube-like morphologies [64]. Here, we employ the same methodology to identify the SAW number and size distribution in quasi-2D, plate-like polymer films. Apart from the direct enumeration for moderately long chains our goal is to provide the scaling exponents in the growth formulas that can be used to predict the SAW behavior as a function of chain size, crystal structure, and level of confinement for significantly longer chains.

The concept of random walk is central to stochastic processes and is applicable to a very wide range of scientific fields and research topics from mathematics, economics, image processing, and social networks to computer science, biology, genetics, and materials [65,66,67,68,69,70,71,72,73,74,75,76,77,78,79,80,81,82,83,84,85,86,87,88,89,90,91]. Self-avoiding random walks have been used extensively to study randomness as observed in kinetics, dynamics, propagation, growth, percolation phenomena and molecular conformations in soft matter [92,93,94,95,96,97,98]. Of particular importance is the SAW model in polymer science as it is directly related to the free-flight models describing chain conformations under various conditions (bulk, confinement, surface adsorption, non-linear chain architecture, chain flexibility, nanofillers etc.) [99,100,101,102,103,104,105,106,107,108,109,110,111,112,113,114,115,116,117,118,119,120,121]. From the technical perspective, since the early work of Orr [112], significant progress has been made towards the development of algorithms that allow efficient SAW enumeration and calculation of the critical exponents in scaling expressions [87,122,123,124,125,126,127,128,129,130,131,132,133]. These are important algorithmic milestones in the SAW enumeration problem that becomes more than exponentially difficult as the number of steps increases.

## 2. Materials and Methods

In a first step, Monte Carlo simulations have been conducted using the algorithm described in Ref. [63] to generate and successively equilibrate freely-jointed chains of tangent hard spheres of uniform size under plate-like confinement (*d*_conf_ = 1). Average chain lengths range from *N*_mon_ = 8 to 1000 and packing densities from *ϕ* = 0.20 to 0.55. We recall here that the freezing and melting points for monomeric hard spheres in the bulk correspond to 0.494 and 0.545, respectively. For chains of tangent hard spheres in the bulk, given the crucial effect of bond tangency/gaps [51,52], the melting point is delayed until a concentration range of *ϕ* ≈ 0.58 is reached [43,44,45,46].

Initial system configurations correspond to cubic cells (*ζ* = 1) under full confinement (*d*_conf_ = 3) which have been generated at dilute conditions and compressed through the wall-displacement (MRoB) algorithm [63] until the desired volume fraction is reached. Then, MRoB is further employed to progressively increase the cell aspect ratio. This process results in the inter-wall distance, *D*_wall_, being reduced until the limit of extreme confinement *D*_wall_ → σ. System configurations are generated at regular intervals during the box transformation phase. Subsequent long MC simulations undertake the task of equilibration with a duration that exceeds hundreds of billions of steps.

As will be demonstrated in the continuation flexible chains, under extreme plate-like confinement, crystallize into well-defined patterns at concentrations significantly lower than the ones in the bulk. Based on this, in the second phase we map the flexible polymer chain onto a SAW on restricted lattice. Here, we follow the original concept presented by Benito et al. [64] according to which in such spatially restricted polymer crystals monomers adopt positions which closely approach the sites of regular lattices. Accordingly, information on the configurational entropy of the freely-jointed chains in plate-like templates can be extracted by analyzing the corresponding SAWs on restricted crystal lattices under the same geometry and conditions (SAW length, lattice type).

We enumerate all possible distinct SAWs on regular lattices corresponding to cubic crystals (SC, BCC and FCC with coordination numbers 6, 8, and 12, respectively). The reference case is the unrestricted one: SAWs on bulk systems under periodic boundary conditions applied in all dimensions. For the bulk lattices and given a specific chain model (i.e., fully flexible one) the number of distinct SAW configurations, *c_N_*, and the average SAW size, as quantified through the mean square end-to-end distance $\langle {\left|\omega^{N}\right|}^{2}\rangle$, depend solely on the number of SAW steps, *N*. However, by introducing plate-like confinement, the spatial group symmetry of the original unrestricted system is reduced from the original Ia$\bar{3}$d. As a result of this, and of the heterogeneity of the confined system, three additional parameters must be considered: level (or intensity) of confinement, the relative orientation of the regular lattice with respect to the axis of confinement, and the initial position (origin) of the SAW, which will be referenced to as “Type” throughout the manuscript. The confinement level can be expressed in terms of the number of crystal layers, *n*, between the parallel plates or as the corresponding inter-plate distance, *D*_wall_, measured in units of the SAW step length.

**Figure 1:**
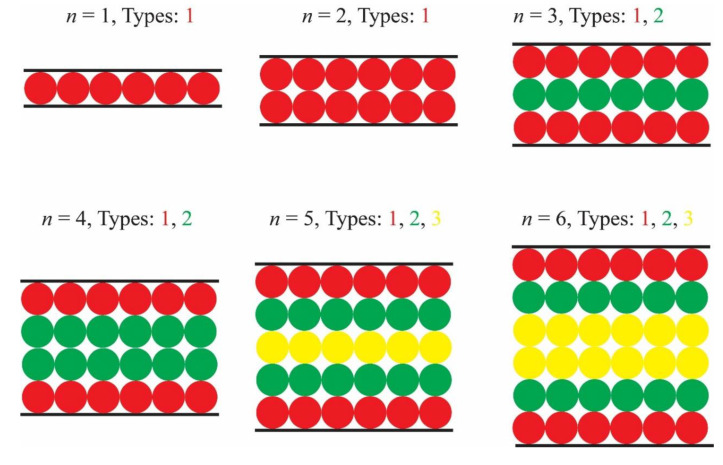
Schematic representation (side view) of distinct origins to be considered for the enumeration of self-avoiding random walks (SAWs) for systems under plate-like confinement. Black lines at the top and bottom parts mark the confining boundaries (plates). Level of confinement is quantified through the number of crystal layers between the plates, *n*, along the axis of confinement. Labeling according to SAW origin depends on the distance from the closest plate. Different color corresponds to different SAW origin (Type). Red, green and yellow colors correspond to Type 1 (closest to the plates), 2 and 3 (furthest from the plates).

The inclusion of the SAW origin (or Type) parameter is a result of the spatial restrictions and the break of symmetry imposed by the plate confinement: the symmetry of Ia$\bar{3}$d of the bulk case is reduced to I4_1_/*acd* due to the presence of the flat, impenetrable walls along the confined dimension. As in Ref. [64] the orientation of the plate axis is defined by direction indices according to the crystallographic practice: [*ijk*]. Given that the crystalline domains are formed with their orientation aligned along the plate section the confining plates are contained in the planes of the crystallographic form [100]. Effectively, a SAW grows on a restricted lattice, RL(*D*_wall_) defined as:

$$RL\left(D\right)=\left\{\underline{x}|\;x_{1},\;x_{2}\in Z,\;\left|x_{3}\right|<D_{\mathrm{wall}}\;\right\}$$

where $\underline{x}$ defines the coordinates of every lattice node and ***Z*** is the unit hypercube of dimension one. Due to the symmetry of the cubic system any axis can be designated as the confined one, denoted in the equation above as *x*_3_. The enumeration process and successive analysis take into account the SAW Type as an additional system parameter: For a given number of steps SAWs starting from origins close to the plate boundaries are expected to show smaller *c_N_* number than the ones growing far from them. Figure 1 shows various cases of plate confinement and the corresponding distinction of lattice sites belonging to different types. For simplicity, a 2-D square lattice is displayed with a varied number of layers, *n*. For the cubic (or square) lattice the number of layers coincides with the inter-plate distance (measured in units of SAW step length). However, this is not the case for the BCC and FCC lattices. Due to symmetry, all nodes that belong to the same layer are characterized by the same Type. Layers are colored according to their Type (SAW origin), which in turn depends on the distance from the closest confining boundary. For example, for *n* = 2, two layers of lattice points exist but both correspond to the same SAW Type as they are similarly adjacent to a different plate wall. The value *n* = 3 leads to two different SAW origins, one in the center and one touching the wall. In general, for the cubic crystal system (SC, BCC and FCC) under plate confinement for even values of the number of layers, *n*, there exist in total *n*/2 distinct SAW Types, while for odd ones the corresponding number changes to (*n* − 1)/2 + 1. In the present work, the assignment of Type starts from the layers adjacent to the walls (Type 1) and ends at the ones in the middle. In Figure 1 for *n* = 6, red (closest to the confining plates), green and yellow (furthest from the confining planes) layers have been assigned Types of 1, 2, and 3, respectively. Multiplicity corresponds to the number of crystallographically equivalent restricted lattices points. For even numbers of *n* all Types have a multiplicity equal to 2, and the same is true for odd numbers with the only exception being the points of the central layer which show cardinality of unity. Multiplicity of SAW Type is important to determine symmetry as it effectively reduces the number of studied systems and the corresponding computational time in SAW enumeration.

**Figure 2:**
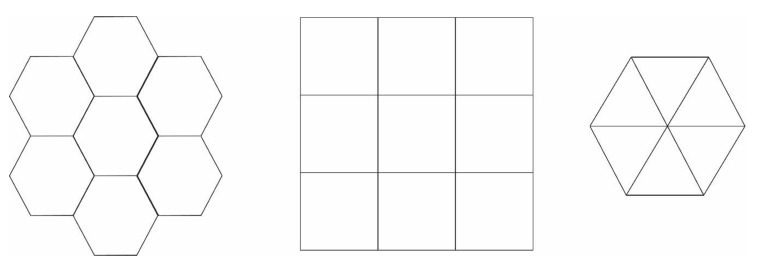
Schematic representation (top view) of the 2-D lattice templates studied here corresponding to extreme thin-film confinement (one layer between confining plates, *n* = 1). From left to right: honeycomb (coordination number of 3), square lattice (coordination number of 4) and triangular (coordination number of 6) lattices.

A distinction between the different lattices of the cubic system can be established once the number of layers between plates becomes equal or exceeds 2. Obviously, the most extreme case corresponds to a single layer under confinement, i.e., *n* = 1. In such 2-D templates the corresponding lattices, studied in the present work, are honeycomb (coordination number of 3), square (coordination number of 4) and triangular (coordination number of 6) as seen in Figure 2.

To summarize, for the given chain architecture (fully flexible linear chains) the number and size of SAWs depend on (i) number of steps, *N*, (ii) lattice type, (iii) level of plate confinement, quantified primarily here through the number of crystal layers between the parallel plates, *n* and (iv) Type (point of SAW origin). The parametric analysis per regular lattice is as follows: SC: *N* ∈ [1, 18], *n* ∈ [1, 5]; BCC: *N* ∈ [1, 15], *n* ∈ [1, 5]; FCC: *N* ∈ [1, 13], *n* ∈ [1, 5]. Obviously, in direct enumeration for a fixed number of SAW steps computational time increases as the coordination number of the lattice increases. Accordingly, the longest chains were accessed for the SC lattice and the shortest SAWs were modeled for the FCC crystal.

In total 376 different 3-D systems were studied: 150 for SC, 117 for BCC and 109 for FCC restricted lattices. In the most extreme case, corresponding to 2-D polymer films, 58 systems were studied: 25 for the honeycomb, 18 for the square, and 15 for the triangular lattices. The main parameters of the modeled systems are reported in Table 1. A home-made SAW code for direct enumeration was developed and all simulations were conducted on Linux-based Intel i7-8700K CPU architectures with 32 Gb of memory.

## 3. Results

### 3.1. Monte Carlo Simulations

**Figure 3:**
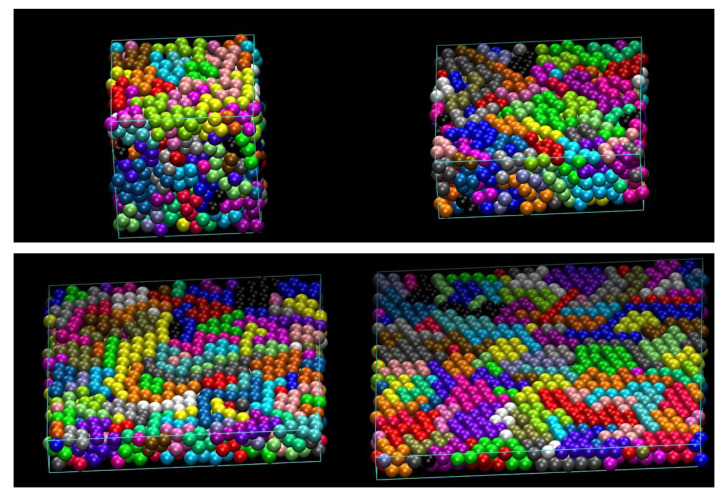
Snapshots of the 100-chain *N*_mon_ = 12 hard sphere system at *ϕ* = 0.50 and under unidimensional confinement (*d*_conf_ = 1) for various cell aspect ratios, *ζ*, corresponding to plate-like geometries. From left to right and from top to bottom: *ζ* = 1 (10.8), 3 (5.2), 7 (3.0) and 12 (2.0). Number in parenthesis indicates the inter-wall distance in the direction of confinement (in units of sphere diameter). Sphere monomers are color-coded according to the parent chain. Image panels created with the VMD software [134]. (please see supplementary materials for 3D version).

Snapshots at the end of the MC equilibration for the *N*_mon_ = 12 system can be seen in Figure 3. The system contains 100 chains with a minimum and maximum chain length of 8 and 16, respectively, at *ϕ* = 0.50 under unidimensional, plate confinement (*d*_conf_ = 1) and for various cell aspect ratios. The packing density *ϕ* = 0.50 of all structures in Figure 3 is well below the transition point for athermal chains in the bulk (*ϕ* ≈ 0.58). Still, as can be seen from a visual inspection of the bottom-right panel of Figure 3, which corresponds to extreme confinement (*ζ* = 12 and *D*_wall_ = 2), monomers on both surfaces show very clear signs of ordering.

**Figure 4:**
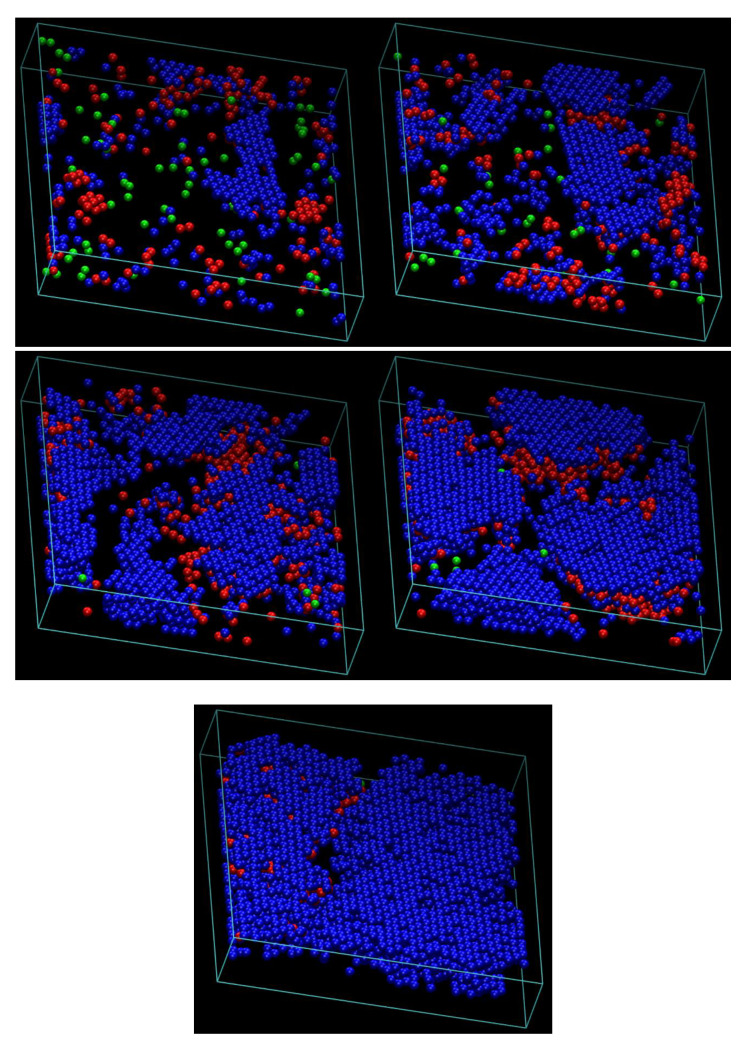
Snapshots of the 10-chain *N*_mon_ = 1000 system at *ϕ* = 0.55 under plate-like confinement with cell anisotropy index, *ζ* = 5 and interwall distance, *D*_wall_ ≈ 7. Top: very early in the simulation (10^9^ MC steps); middle: (left) 2 × 10^10^ and 5 × 10^10^ (right) MC steps; bottom: 12 × 10^10^ (left) and at the end of simulation, 14 × 10^11^ (right) MC steps. Monomers with HCP, FCC and fivefold similarity as identified by the CCE norm, are shown in blue, red and green, respectively. All other, “amorphous” sites do not appear for clarity. Image created with the VMD software [134]. (please see supplementary materials for 3D version).

Crystal nucleation and growth can be accurately identified and then quantified by applying the characteristic crystallographic element (CCE) norm, which is able to distinguish between different competing crystal structures [44,135,136]. As the athermal chain packings correspond to high concentration, we employ the CCE norm with respect to the FCC and HCP crystals as well as the fivefold local symmetry. The CCE norm is applied on all sites/monomers present in the system. Once the value of the CCE norm with respect to a specific crystal *X*, *ε^X^*, is lower than a critical threshold (*ε^X^* < 0.245), the site is identified as of *X* similarity. Due to the distinguishing nature of the crystallographic elements, no site can possess dual crystal similarity. Figure 4 hosts configurations, as the MC simulation evolves, for the 10-chains *N*_mon_ = 1000 system at *ϕ* = 0.55 (still quite below the bulk melting point of athermal polymers), showing only monomers with HCP (blue), FCC (red) and fivefold (green) local environment. All other sites, labeled as “amorphous”, are not shown for clarity purposes. Starting from the initially amorphous state (upper panel), the system shows a clear disorder–order transition with the stable crystal increasing in size as the observation time increases. The final stable configuration is highly close packed with predominant HCP character.

### 3.2. Verification with Available Literature Data

As mentioned in Section 2, centers of the spherical monomers adopt positions that resemble closely nodes of a perfect crystal. For example, this tendency is particularly evident for the case shown in the bottom-right panel of Figure 3. Thus, we model linear flexible polymers in confined space as self-avoiding random walks on restricted lattices.

First, results are compared against literature data on the well-studied SAWs on bulk 3-D SC, BCC and FCC lattices and on most extremely confined ones that correspond to 2-D lattices (HON, SQU and TRI). The unrestricted (bulk) case can be modeled either by removing any spatial conditions related to confinement or by having the number of lattice layers, *n*, to be larger than the maximum possible chain extension, i.e. *n* > *N* + 1. Data on the number of distinct SAWs, *c_N_*, and on the mean square end-to-end distance, $\langle {\left|\omega^{N}\right|}^{2}\rangle$, as a function of SAW steps, *N*, can be found in Table A1 and Table A2 of the Appendix A for the extremely confined 2-D and the unrestricted 3-D lattices, respectively. For all regular lattices studied here extreme confinement (*n* = 1) involves a single SAW origin (Type 1); the same is true for the 3-D bulk cases due to symmetry considerations. Results for the bulk SC and FCC lattices are in perfect quantitative agreement with our past work conducted through a different numerical algorithm [64]. Furthermore, for both SAW populations and average sizes our enumeration data coincide with the ones in Refs. [131] and [132] for SC; in Ref. [133] for bulk BCC and FCC; in Ref. [137] for the 2-D triangular and in Ref. [128] for square, honeycomb, and SC lattices. We should note here that, given the large number of systems to be studied (three different lattice types, different Types and levels of confinement), our goal is not to exceed or even reach the current state-of-the-art in modeled SAW lengths but rather to establish asymptotic scaling formulas for the confined cases. These will allow us to predict the behavior of long SAWs (and equivalently of long chains) from results on short or intermediate ones and establish a systematic connection between plate-like confinement and properties of the corresponding SAWs. Table A3, Table A4, Table A5, Table A6, Table A7, Table A8, Table A9, Table A10, Table A11 and Table A12 host the properties of SAWs (*c_N_* and $\langle {\left|\omega^{N}\right|}^{2}\rangle$) for all confined lattices with the number of layers between plates lying in the interval *n* ∈ [2, 5]. An interesting trend can be observed for the BCC lattice with *n* = 2 (Table A3): the number of distinct SAWs coincides with the one extracted for the SQU lattice (Table A1). This is because with respect to connectivity there is no distinction between the square and the 2-layer BCC lattices.

### 3.3. Direct SAW Enumeration

Data (*c_N_* vs. *N* and $\langle {\left|\omega^{N}\right|}^{2}\rangle$ vs. *N*) as presented in Table A1, Table A2, Table A3, Table A4, Table A5, Table A6, Table A7, Table A8, Table A9, Table A10, Table A11 and Table A12, obtained from SAWs of short to intermediate length, can be used in the asymptotic formulas [131,133,137] for the scaling of the number of distinct SAWs in the limit of *N* → ∞:

(1) $$c_{N}\sim A\mu^{N}N^{\gamma -1}$$

and of the mean-square, end-to-end distance:

(2) $$\langle {\left|\omega^{N}\right|}^{2}\rangle \sim DN^{2v}$$

where *γ* and *υ* are the critical exponents, *A* and *D* are the critical amplitudes, and *μ* is the connective constant. While *A*, *D,* and *μ* depend on lattice type the critical exponents *γ* and *υ* are considered universal [133,137,138,139]. As proven by Duminil-Copin and Smirnov [140] the connective constant for the honeycomb lattice is equal to $\mu =\sqrt{2+\sqrt{2}}$, as originally conjectured by Nienhuis [141,142].

**Figure 5:**
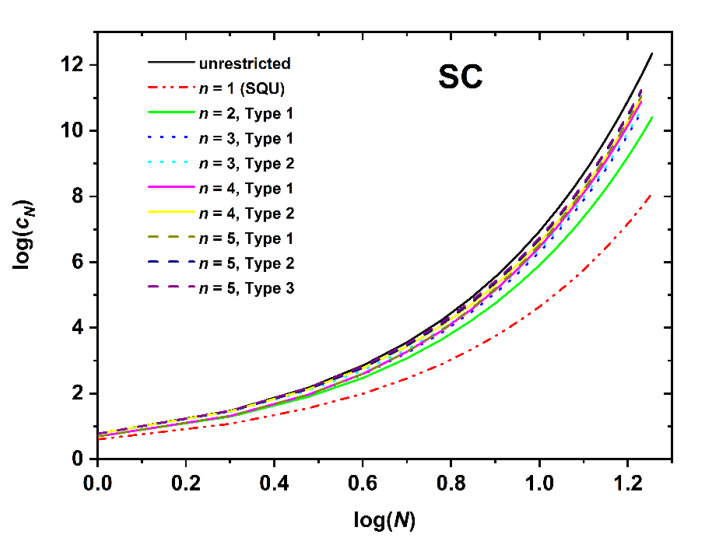
Logarithm of the number of distinct SAWs, *c_N_*, versus the logarithm of the number of SAW steps, *N*, as obtained from direct enumeration on the SC lattice under plate-like confinement. n corresponds to the number of layers between the confining plates. Label “Type” corresponds to different SAW origin as explained in Figure 1 and related text. The most extremely confined case of *n* = 1 corresponds to the 2-D square lattice (SQU).

**Figure 6:**
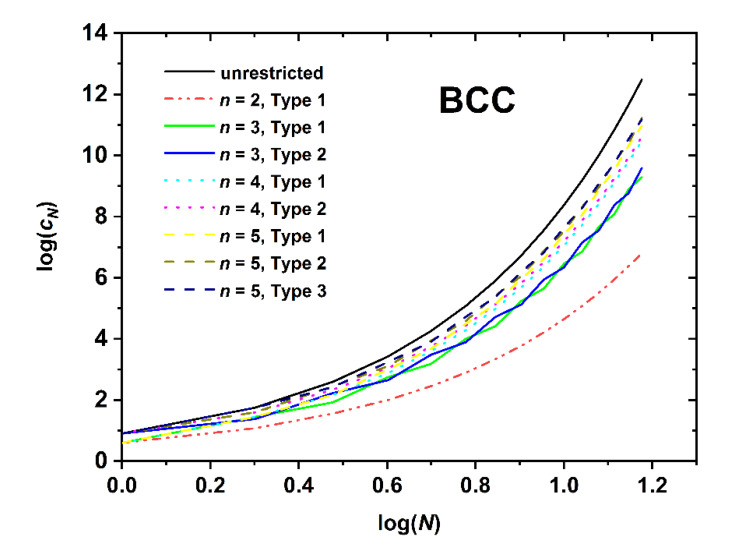
Logarithm of the number of distinct SAWs, *c_N_*, versus the logarithm of the number of SAW steps, *N*, as obtained from direct enumeration on the BCC lattice under plate-like confinement.

**Figure 7:**
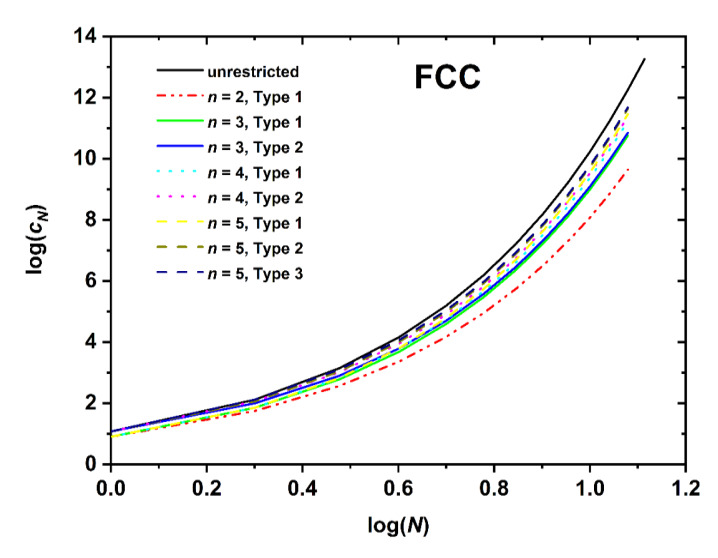
Logarithm of the number of distinct SAWs, *c_N_*, versus the logarithm of the number of SAW steps, *N*, as obtained from direct enumeration on the FCC lattice under plate-like confinement.

Figure 5, Figure 6 and Figure 7 present the logarithm of the number of distinct SAWs versus the logarithm of the number of SAW steps for the restricted SC, BCC, and FCC lattices, respectively. For all systems studied here, the combination of SAW size (chain length) and applied confinement force the self-avoiding random walks to “feel” the imposed spatial constraints. In all cases the obvious trend is fully established: the stronger the restrictions imposed by the film-like confinement the smaller the number of available SAWs, or equivalently, the fewer the number of distinct chain configurations and accordingly reduced configurational entropy. The most extremely confined system (*n* = 1 for SC (SQU) or *n* = 2 for BCC and FCC) is the one that deviates markedly from the unrestricted bulk case. As film thickness (inter-plate distance) increases SAW properties converge to the ones in the bulk. Between different Types, the lattice nodes and layers lying closer to the confining surfaces are characterized systematically by smaller *c_N_* than the ones near the center. This is manifestly valid for the SC and FCC lattices, but not always true for the BCC, as can be readily observed by comparing the different Type columns in the Appendix A Tables. This difference in trends can be explained by the coordination number which remains the same for SC but depends on the layer index for BCC. In general, the effect of SAW origin (Type) on SAW properties is smaller in plate-like (quasi 2-D) confinement, as established here, than in tube-like (quasi 1-D) restricted lattices, as reported in Ref. [64].

Comparing the different restricted lattices of the cubic system trends analogous to the bulk case are established as the number of distinct SAWs increases significantly with the coordination number. For example, for a fixed number of SAW steps (*N* = 12), film thickness (*n* = 3), and SAW origin (Type 1), SAW population starts from *c_N_* = 33,574,732 (SC), increases to 47,788,288 (BCC), and end ups at 56,963,463,220 (FCC); an increment that spans three orders of magnitude for identical conditions of spatial restriction and which can be purely attributed to the increase in coordination number.

**Figure 8:**
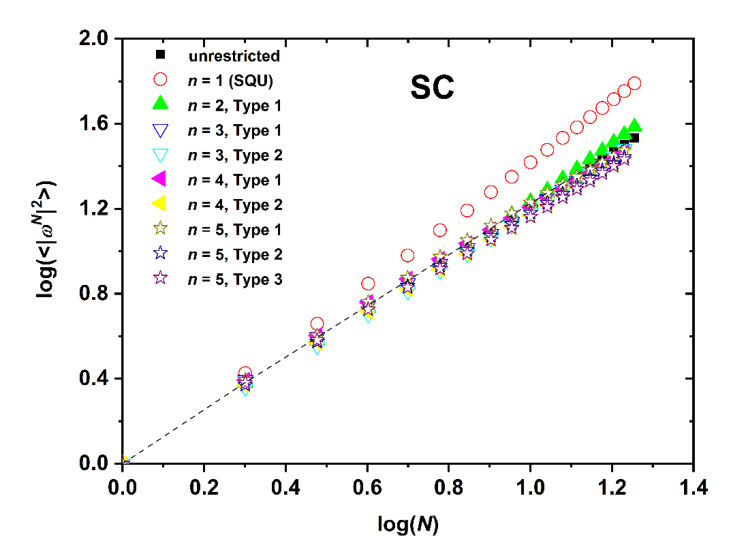
Logarithm of the mean square end-to-end SAW distance, $\langle {\left|\omega^{N}\right|}^{2}\rangle$, as a function of the logarithm of SAW steps, *N*, as obtained for the SC lattice under plate-like confinement. *n* corresponds to the number of layers between the confining plates. Label “Type” corresponds to different SAW origin as explained in Figure 1 and related text. The limiting case of *n* = 1 corresponds to the 2-D square lattice (SQU). Dashed black line corresponds to best linear fit on bulk SAW data.

**Figure 9:**
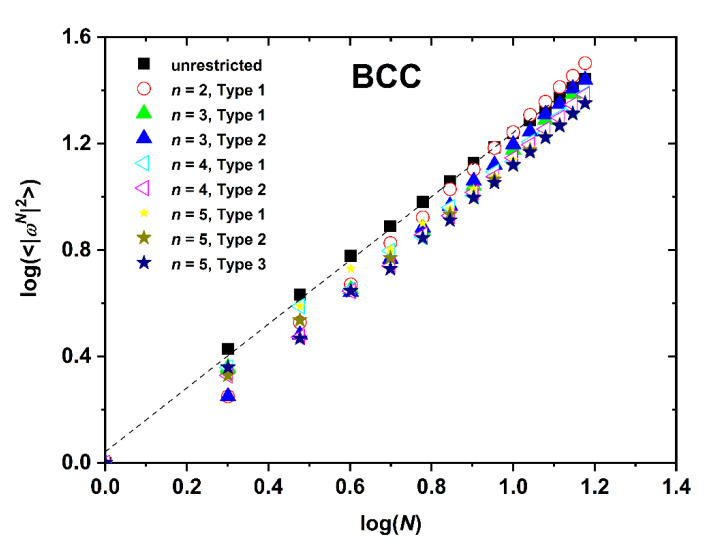
Logarithm of the mean square end-to-end SAW distance, $\langle {\left|\omega^{N}\right|}^{2}\rangle$, as a function of the logarithm of SAW steps, *N*, as obtained for the BCC lattice under plate-like confinement. Dashed black line corresponds to best linear fit on the bulk case over the whole range of available data.

**Figure 10:**
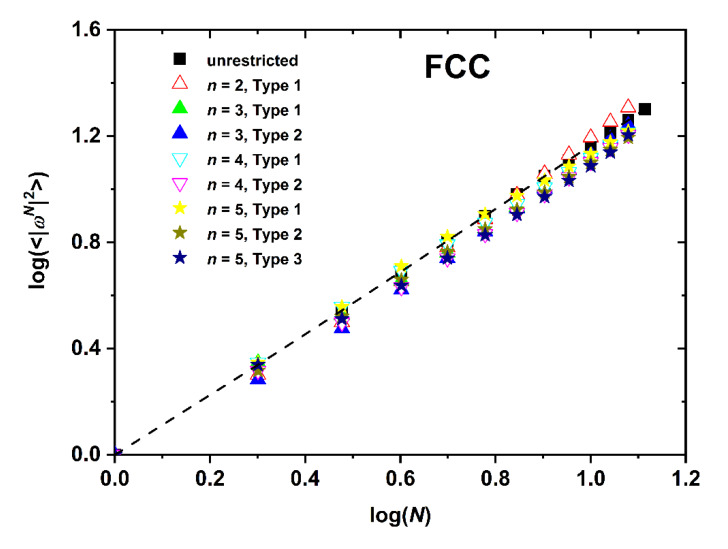
Logarithm of the mean square end-to-end SAW distance, $\langle {\left|\omega^{N}\right|}^{2}\rangle$, as a function of the logarithm of SAW steps, *N*, as obtained for the FCC lattice under plate-like confinement. Dashed black line corresponds to best linear fit on the bulk case over the whole range of available data.

The dependence of SAW size, as quantified by the mean square end-to-end distance, on the number of SAW steps is presented in Figure 8, Figure 9 and Figure 10 for the SC, BCC, and FCC restricted lattices, respectively.

Based on the log(*c_N_*)-*vs*.-log(*N*) (Figure 5, Figure 6 and Figure 7) and the log($\langle {\left|\omega^{N}\right|}^{2}\rangle$)-*vs*.-log(*N*) (Figure 8, Figure 9 and Figure 10), non-linear fits on the growth formulas in Equations (1) and (2) yield all critical parameters (*A*, *D*, *μ*, *γ* and *ν*). Results from such statistical analysis can be found in Table 2, Table 3 and Table 4 for the restricted SC, BCC, and FCC lattices, respectively. In all cases data are compared with the reference bulk crystal. The connectivity constant, *μ*, decreases as the spatial restriction becomes stronger. It adopts the lowest value for the most extremely confined system and as the number of layers increases it progressively converges to the limiting value of the bulk counterpart. Compared to the connectivity constant the critical amplitude, *A* and the exponent *γ* depend rather weakly on level of confinement and SAW origin. Under the same conditions of confinement, connectivity constant increases as the lattice coordination number increases (*μ*_SC_ < *μ*_BCC_ < *μ*_FCC_). In general, for SC and FCC restricted lattices, for layers closer to the confining agents, *μ* is higher than for layers near the center, i.e., the connectivity constant decreases with increasing Type index for SC and FCC while the opposite trend is observed for the BCC lattice.

**Figure 11:**
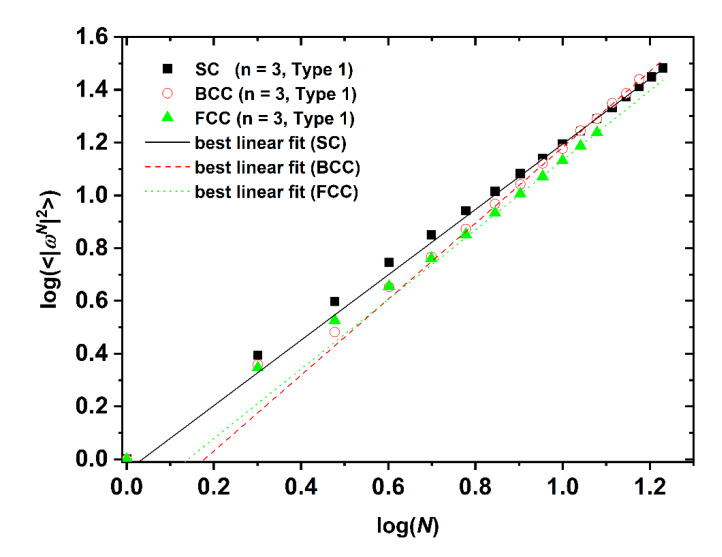
Logarithm of the mean square end-to-end SAW distance, $\langle {\left|\omega^{N}\right|}^{2}\rangle$, as a function of logarithm of number of SAW steps for the SC, BCC and FCC lattices under plate confinement (*n* = 3, Type 1). Also shown are the lines that correspond to best linear fits in the large-N data range once normal scaling has been established.

The SAW generating function (Equation (1)) is valid for the whole range of available data, independently of dimensionality, lattice type, level of confinement, and point of origin (Type). However, the same is not true for the dependence of SAW size on number of SAW steps (Equation (2)). While the unrestricted lattices show linear scaling all confined ones at short-*N* deviate significantly from linearity. Such trends have also been observed in the SAW analysis of restricted lattices under tube-like confinement [64]. The larger the lattice coordination number and the closer to the confining plates (low Type index), the most prolonged the duration of the anomalous regime, as can be seen in Figure 11 where the SAW size evolution is presented for the SC, BCC, and FCC lattices with *n* = 3 and Type 1.

Based on the parameters extracted from the linear fitting in the range of large-*N* data the following conclusions can be established: in general, the critical exponent, *v*, adopts its maximum value under the most confined case while its minimum corresponds to the unrestricted (bulk) case. All confined systems are characterized by amplitude values *D* which are significantly different than the ones of the bulk lattice. For restricted SC and FCC lattices, SAW origin (practically the distance of the layer from the confining agents) has an appreciable effect on *D* and *v* values. As Type index increases *D* decreases appreciably and the opposite trend is observed for *v*. The behavior of the BCC restricted lattice does not follow the trends of the other two crystals. Accordingly, no systematic behavior can be identified for BCC crystal.

### 3.4. SAW Size Distribution

**Figure 12:**
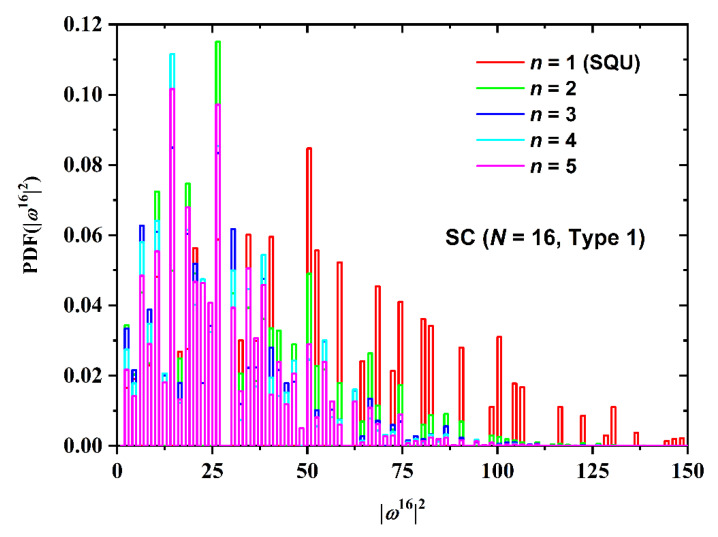
Probability distribution function (PDF) of the square end-to-end distance for the SC restricted lattice for SAWs of fixed length (*N* = 16) and point of origin (Type 1). Different curves correspond to differ number of layers between the confining plates, *n*. The case of *n* = 1 corresponds to the 2-D square lattice (SQU).

Information is also available not only on the average SAW size but also on the probability distribution function (PDF) and cumulative distribution function (CDF) as a function of lattice type, level of confinement and SAW origin (Type). Additional information can be extracted from the analysis of the folded CDF variant focusing on the median and the corresponding deviation. Given the plethora of systems studied and due to space limitations, in the following only selected systems are presented for the size distribution. Figure 12 shows the PDF of size as a function of the number of layers between confinement, *n*, for the restricted SC having fixed *N* = 16 and Type 1. As stated before, the case of *n* = 1 corresponds to the 2-D square lattice. The number of confined layers has a significant effect on the size distribution: the most extreme confinement (*n* = 1) and the least confined (*n* = 5) cases correspond to the broadest and narrowest distributions, respectively. In general, as confinement increases, the size distribution becomes broader and shifts to higher values.

**Figure 13:**
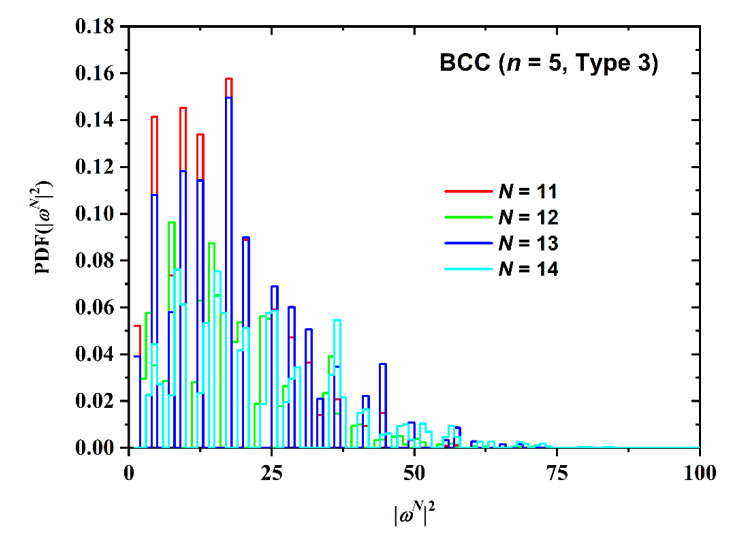
Probability distribution function (PDF) of the square end-to-end distance for the BCC restricted lattice for SAWs of fixed number of layers between confining plates (*n* = 5) and point of origin (Type 3). Different curves correspond to different number of SAW steps, *N*.

**Figure 14:**
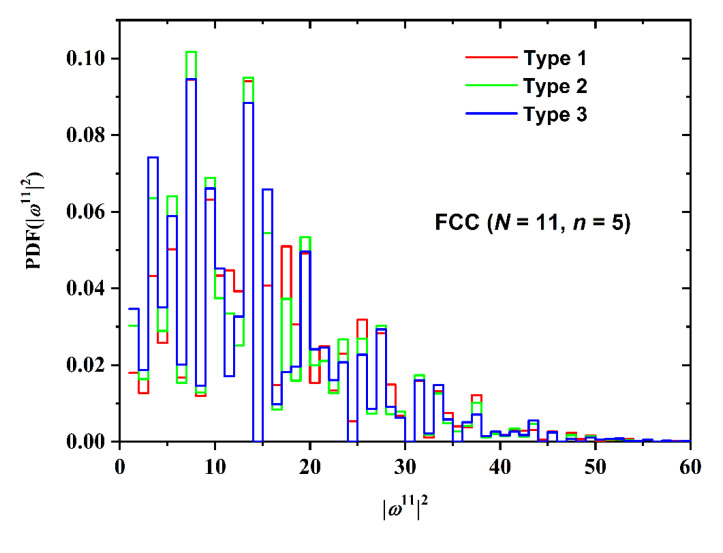
Probability distribution function (PDF) of the square end-to-end distance for the FCC restricted lattice for SAWs of fixed length (*N* = 11) and number of layers between confining plates (*n* = 5). Different curves correspond to different point of origin (Type).

Not surprisingly, the number of SAW steps has a stronger effect on the SAW size distribution as seen in Figure 13 for the BCC restricted lattice (*n* = 5, Type 3). As chain length increases the distribution becomes broader, it shifts to higher values and the corresponding maxima get significantly reduced. The effect of point of SAW origin (Type) on the SAW distribution is presented in Figure 14 for the FCC lattice (*N* = 11, *n* = 5). For up to two layers in plate-like confinement there is no distinction in SAW Type. Based on the results in Figure 14 it can be concluded that Type (in other words the starting layer) has a minor effect on SAW size, which is further diminishing as the number of SAW steps increases.

**Figure 15:**
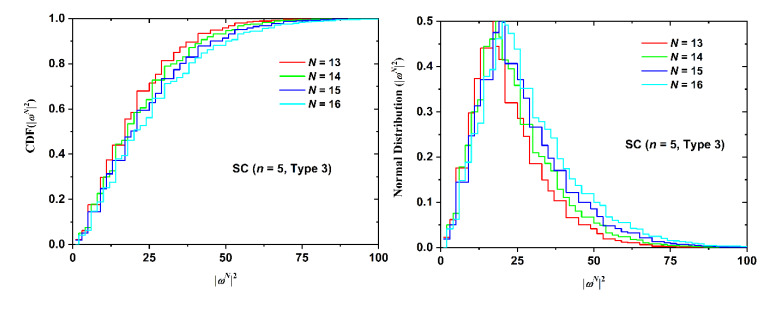
(Left Panel) Cumulative distribution function (CDF) and (Right Panel) the folded variant of the square end-to-end distance for the SC restricted lattice for SAWs of fixed number of confined layers (*n* = 5) and point of origin (Type 3). Different curves correspond to different number of SAW steps, *N.*

**Figure 16:**
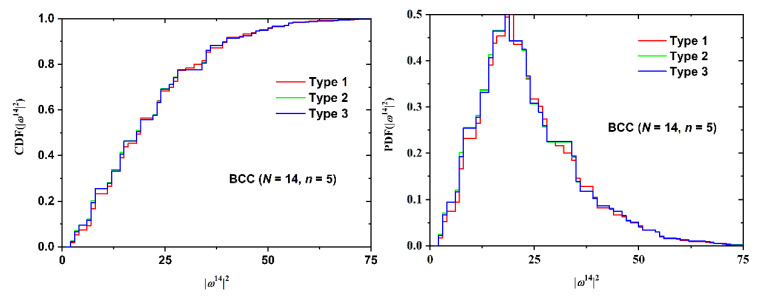
(Left Panel) Cumulative distribution function (CDF) and (Right Panel) the folded variant of the square end-to-end distance for the BCC restricted lattice for SAWs of fixed length (*N* = 14) and number of layers between confining planes (*n* = 5). Different curves correspond to different SAW origins (Types).

**Figure 17:**
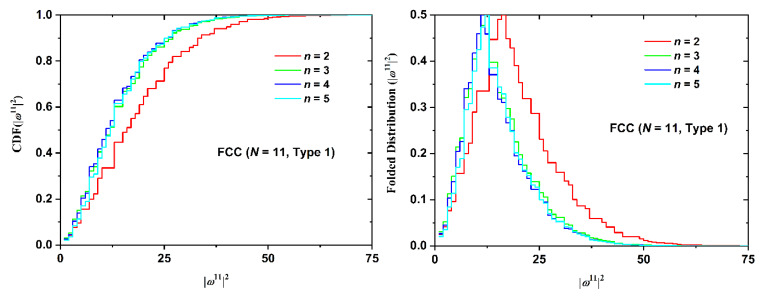
(Left Panel) Cumulative distribution function (CDF) and (Right Panel) the folded variant for the square end-to-end distance for the FCC restricted lattice for SAWs of fixed length (*N* = 11) and point of origin (Type 1). Different curves correspond to different number of layers between the confining plates, *n*.

Cumulative distribution functions and the folded variants for SAW size, as quantified by the square end-to-end distance, are presented in Figure 15 (SC lattice with fixed *n = 5*, Type 3 and varied *N*), Figure 16 (BCC lattice with fixed *N* = 14, *n* = 5 and varied Type), and Figure 17 (FCC lattice with fixed *N* = 11, Type 1 and varied *n*). 

The same conclusions can be drawn from the data of the cumulative and folded distributions. As the number of SAW steps increases, the distribution of size becomes broader, shifts to higher values, and the intensity of the observed maxima drops. SAW origin has a minor effect on size statistics. The inter-plate thickness has the strongest effect as the more confined the polymer chain, the more extended it becomes. The statistics of the selected folded distributions (most probable, median, and deviation values) can be found in Table A13, Table A14, Table A15, Table A16, Table A17 and Table A18 of the Appendix A. All lattice types show identical trends, especially the strong dependence on film thickness and the very weak one on SAW origin, as validated by the comparison of the mean value and the corresponding deviation.

## 4. Conclusions and Future Plans

In the present contribution, we have studied the behavior of athermal polymer chains under extreme confinement realized through the presence of parallel, flat, and impenetrable walls in one dimension. The inter-plate distance is so small that it practically adopts values similar to the size of the spherical monomers. Presently, Monte Carlo simulations show that dense packings of highly confined chains tend to crystallize at volume fractions which are significantly lower than the corresponding threshold of the bulk case. In an effort to identify the thermodynamic stability of the corresponding structures and the entropic origins of the phase transitions, we have mapped the athermal chains onto self-avoiding random walks (SAWs) on lattices which are further spatially confined as the atomistic analogs. Given that the applied confinement breaks the original maximal symmetry of the original crystal, we have analyzed the effect of number of SAW steps (chain length), the level of confinement (film thickness quantified by the number of lattice layers between the plates), and point of origin on the size of chains and on their number of distinct conformations. The latter is important as it is directly related to the configurational entropy of the chains.

The present work on plate-like (quasi 2-D) confinement, as well as the past on tube-like (quasi 1-D) restrictions [64], constitute the first step of an ongoing research effort. The final goal is to investigate and predict phase (disorder-order) transitions in confined athermal polymeric systems, for which entropy is the sole driving force. The entropy of the freely-jointed chains in plate-like templates can be obtained by direct enumeration of SAWs on restricted crystal lattices under the same geometry and conditions (SAW length, lattice type).

## Figures and Tables

**Table 1:** Regular lattices studied in three dimensions (simple cubic (SC), body centered cubic (BCC) and face centered cubic, (FCC)), and in two dimensions (honeycomb (HON), square (SQU) and triangular (TRI)). Also shown are the number of layers, *n*, and the distance, *D*_wall_, between the confining plates and the different SAW Types (points of origin). Inter-plate distance is measured in units of the SAW step length.

Lattice Type	Number of Layers between Plates, *n*	Distance between Plates, *D*_wall_	Type (SAW Origin)
SC	2	1	1
	3	2	1, 2
	4	3	1, 2
	5	4	1, 2, 3
BCC	2	$1/\sqrt{3}$	1
	3	$2/\sqrt{3}$	1, 2
	4	$3/\sqrt{3}$	1, 2
	5	$4/\sqrt{3}$	1, 2, 3
FCC	2	$1/\sqrt{2}$	1
	3	$\sqrt{2}$	1, 2
	4	$3/\sqrt{2}$	1, 2
	5	$2\sqrt{2}$	1, 2, 3
HON	1	0	1
SQU	1	0	1
TRI	1	0	1

**Table 2:** Critical parameters of the asymptotic formulas (Equations (1) and (2)) as obtained from non-linear fits on the SAW data presented in Figure 5 and Figure 8 for the confined SC lattice. Also shown for comparison are the results for the bulk (unrestricted) SC.

*N*	Type	*A*	*μ*	*γ*	*D*	*υ*
2	1	1.375	3.622	1.181	0.958	0.631
3	1	1.214	4.090	1.097	0.903	0.619
	2	1.515	3.963	1.236	0.761	0.650
4	1	1.137	4.373	0.988	1.267	0.552
	2	1.430	4.188	1.226	0.942	0.598
5	1	1.102	4.523	0.919	1.445	0.535
	2	1.377	4.353	1.165	1.103	0.569
	3	1.387	4.308	1.258	0.983	0.585
Bulk		1.270	4.717	1.103	1.026	0.607

**Table 3:** Critical parameters of the asymptotic formulas (Equations (1) and (2)) as obtained from non-linear fits on the SAW data presented in Figure 6 and Figure 9 for the confined BCC lattice. Also shown for comparison are the results for the bulk (unrestricted) case.

*N*	Type	*A*	*μ*	*γ*	*D*	*υ*
2	1	1.480	2.658	1.228	0.625	0.725
3	1	1.087	3.765	1.559	0.553	0.720
	2	1.783	4.326	0.849	0.597	0.709
4	1	0.888	4.548	1.527	0.602	0.682
	2	1.634	4.860	1.116	0.603	0.682
5	1	0.782	5.133	1.384	0.847	0.605
	2	1.455	5.420	1.058	0.662	0.651
	3	1.580	5.115	1.308	0.641	0.656
Bulk		1.214	6.580	1.108	1.101	0.600

**Table 4:** Critical parameters of the asymptotic formulas (Equations (1) and (2)) as obtained from non-linear fits on the SAW data presented in Figure 7 and Figure 10 for the confined FCC lattice. Also shown for comparison are the results for the bulk (unrestricted) case.

*N*	Type	*A*	*μ*	*γ*	*D*	*υ*
2	1	1.358	5.888	1.257	0.606	0.707
3	1	1.088	7.346	1.307	0.653	0.659
	2	1.605	7.473	1.163	0.604	0.676
4	1	0.941	8.462	1.145	0.889	0.586
	2	1.468	8.178	1.233	0.687	0.634
5	1	0.869	9.171	1.010	1.217	0.525
	2	1.356	8.836	1.148	0.860	0.584
	3	1.425	8.439	1.359	0.625	0.649
Bulk		1.191	10.08	1.129	0.975	0.587

## Supplementary Materials

The following are available online at https://www.mdpi.com/2073-4360/12/4/799/s1: An interactive, 3-D version of the manuscript; 3-D versions of Figure 3 and Figure 4; 3-D video showing animated the panels of Figure 4.

## Abbreviations

The following abbreviations have been used in this manuscript:

MC	Monte Carlo
MD	Molecular Dynamics
SAW	Self-Avoiding Random Walk
CCE	Characteristic Crystallographic Element (norm)
SC	Simple Cubic
BCC	Body Center Cubic
FCC	Face Center Cubic
PDF	Probability Distribution Function
CDF	Cumulative Distribution Function
HON	Honeycomb
SQU	Square
TRI	Triangular

## Appendix A

In the following Tables, we present the number of distinct configurations, *c_N_*, and the mean square end-to-end distance, $\langle {\left|\omega^{N}\right|}^{2}\rangle$, for the self-avoiding random walks (SAWs) as a function of the number of SAW steps, *N*, under plate-like confinement. System parameters include lattice type, number of layers between confining plates, *n*, and Type (SAW origin). Also shown for comparison are the results obtained from simulations on corresponding 2-D lattices (honeycomb (HON), square (SQU) and triangular (TRI)) corresponding to extreme (single-layer) confinement.

**Table A1:** Properties of SAWs on 2-D regular lattices corresponding to extreme, plate-like confinement: honeycomb (HON), square (SQU) and triangular (TRI). In all cases *n* = 1 and Type 1.

	HON (*n* = 1, Type 1)		SQU (*n* = 1, Type 1)		TRI (*n* = 1, Type 1)	
*N*	*c_N_*	$\langle {\left|\omega^{N}\right|}^{2}\rangle$	*c_N_*	$\langle {\left|\omega^{N}\right|}^{2}\rangle$	*c_N_*	$\langle {\left|\omega^{N}\right|}^{2}\rangle$
1	3	1.000	4	1.000	6	1.000
2	6	3.000	12	2.667	30	2.400
3	12	5.500	36	4.556	138	4.217
4	24	8.250	100	7.040	618	6.350
5	48	11.13	284	9.563	2730	8.741
6	90	15.00	780	12.57	11,946	11.36
7	174	18.69	2172	15.56	51,882	14.20
8	336	22.50	5916	19.01	224,130	17.24
9	648	26.42	16,268	22.41	964,134	20.47
10	1218	31.18	44,100	26.24	4,133,166	23.87
11	2328	35.59	120,292	30.02	17,668,938	27.43
12	4416	40.34	324,932	34.19	75,355,206	31.16
13	8388	45.14	881,500	38.30	320,734,686	35.03
14	15,780	50.49	2,374,444	42.79	1,362,791,250	39.06
15	29,892	55.59	6,416,596	47.22	5,781,765,582	43.22
16	56,628	61.13	17,245,332	51.99		
17	106,200	66.60	46,466,676	56.72		
18	199,350	72.53	124,658,732	61.77		
19	375,504	78.29				
20	704,304	84.46				
21	1,323,996	90.53				
22	2,479,692	97.01				
23	4,654,464	103.4				
24	8,710,212	110.1				
25	16,328,220	116.7				

**Table A2:** Number of distinct configurations of SAWs on 3-D regular lattices without spatial restrictions (bulk case): simple cubic (SC), body center cubic (BCC) and face center cubic (FCC). Due to symmetry all nodes correspond to a single SAW origin (Type 1).

	SC (bulk, Type 1)	BCC (bulk, Type 1)	FCC (bulk, Type 1)
*N*	*c_N_*	*c_N_*	*c_N_*
1	6	8	12
2	30	56	132
3	150	392	1404
4	726	2648	14,700
5	3534	17,960	152,532
6	16,926	120,056	1,573,716
7	81,390	804,824	16,172,148
8	387,966	5,351,720	165,697,044
9	1,853,886	35,652,680	1,693,773,924
10	8,809,878	236,291,096	17,281,929,564
11	41,934,150	1,568,049,560	176,064,704,412
12	198,842,742	10,368,669,992	1,791,455,071,068
13	943,974,510	68,626,647,608	18,208,650,297,396
14	4,468,911,678	453,032,542,040	
15	21,175,146,054	2,992,783,648,424	
16	100,121,875,974		
17	473,730,252,102		
18	2,237,723,684,094		

**Table A3:** Properties of SAWs on the SC, BCC and FCC lattices with two layers between the confining plates (*n* = 2, Type 1).

	SC (*n* = 2, Type 1)		BCC (*n* = 2, Type 1)		FCC (*n* = 2, Type 1)	
*N*	*c_N_*	$\langle {\left|\omega^{N}\right|}^{2}\rangle$	*c_N_*	$\langle {\left|\omega^{N}\right|}^{2}\rangle$	*c_N_*	$\langle {\left|\omega^{N}\right|}^{2}\rangle$
1	5	1.000	4	1.000	8	1.000
2	20	2.400	12	1.778	56	2.000
3	80	3.800	36	3.370	368	3.152
4	304	5.421	100	4.693	2336	4.510
5	1168	7.000	284	6.709	14,576	6.037
6	4348	8.854	780	8.383	89,928	7.713
7	16,336	10.68	2,172	10.70	550,504	9.523
8	60,208	12.77	5916	12.68	3,349,864	11.46
9	223,352	14.83	16,268	15.27	20,290,360	13.51
10	817,852	17.14	44,100	17.50	122,445,504	15.68
11	3,008,872	19.43	120,292	20.35	736,685,008	17.95
12	10,968,400	21.94	324,932	22.79	4,421,048,016	20.32
13	40,123,760	24.42	881,500	25.87		
14	145,783,980	27.12	2,374,444	28.52		
15	531,100,496	29.78	6,416,596	31.81		
16	1,924,770,512	32.65				
17	6,990,248,624	35.49				
18	25,282,157,540	38.52				

**Table A4:** Properties of SAWS on SC lattice with 3 layers between the confining plates (*n* = 3, Type 1, 2).

SC (*n* = 3, Type 1)			SC (*n* = 3, Type 2)	
*N*	$c_{N}$	$\langle {\left|\omega^{N}\right|}^{2}\rangle$	$c_{N}$	$\langle {\left|\omega^{N}\right|}^{2}\rangle$
1	5	1.000	6	1.000
2	21	2.476	28	2.286
3	92	3.957	124	3.581
4	392	5.571	516	5.054
5	1684	7.090	2156	6.492
6	7036	8.748	8804	8.125
7	29,396	10.35	36,388	9.717
8	120,776	12.10	148,452	11.50
9	497,196	13.81	609,812	13.25
10	2,026,220	15.68	2,478,484	15.19
11	8,278,076	17.53	10,113,692	17.10
12	33,574,732	19.53	40,934,604	19.18
13	136,456,380	21.52	166,170,388	21.23
14	551,445,764	23.67	670,410,548	23.44
15	2,232,227,600	25.81	2,711,129,404	25.63
16	8,995,089,168	28.09	10,911,074,820	27.97
17	36,297,709,788	30.36	43,995,500,972	30.28

**Table A5:** Properties of SAWs on SC lattice with four layers between the confining plates (*n* = 4, Type 1, 2).

SC (*n* = 4, Type 1)			SC (*n* = 4, Type 2)	
*N*	$c_{N}$	$\langle {\left|\omega^{N}\right|}^{2}\rangle$	$c_{N}$	$\langle {\left|\omega^{N}\right|}^{2}\rangle$
1	5	1.000	6	1.000
2	21	2.476	29	2.345
3	93	4.011	136	3.706
4	408	5.745	604	5.205
5	1832	7.410	2676	6.644
6	8084	9.192	11,564	8.218
7	35,752	10.88	50,228	9.737
8	155,756	12.65	215,492	11.39
9	677,856	14.34	929,136	13.01
10	2,920,764	16.11	3,972,948	14.76
11	12,582,860	17.84	17,048,772	16.48
12	53,858,044	19.65	72,685,616	18.33
13	230,643,688	21.45	310,668,724	20.16
14	983,162,808	23.34	1,320,897,848	22.12
15	4,193,819,200	25.22	5,626,979,444	24.06
16	17,824,575,272	27.20	23,868,686,764	26.12
17	75,809,092,412	29.17	101,405,080,196	28.15

**Table A6:** Properties of SAWs on SC lattice with 5 layers between the confining planes (*n* = 5, Type 1, 2, 3).

SC (*n* = 5, Type 1)			SC (*n* = 5, Type 2)		SC (*n* = 5, Type 3)	
*N*	$c_{N}$	$\langle {\left|\omega^{N}\right|}^{2}\rangle$	$c_{N}$	$\langle {\left|\omega^{N}\right|}^{2}\rangle$	$c_{N}$	$\langle {\left|\omega^{N}\right|}^{2}\rangle$
1	5	1.000	6	1.000	6	1.000
2	21	2.476	29	2.345	30	2.400
3	93	4.011	137	3.745	148	3.811
4	409	5.770	620	5.329	692	5.318
5	1852	7.514	2824	6.875	3196	6.747
6	8308	9.431	12,616	8.547	14,324	8.275
7	37,620	11.29	56,668	10.14	64,076	9.748
8	168,768	13.23	251,500	11.83	282,716	11.33
9	758,340	15.09	1,119,212	13.46	1,251,044	12.88
10	3,379,476	16.98	4,939,768	15.19	5,493,804	14.54
11	15,051,324	18.80	21,845,116	16.86	24,207,436	16.17
12	66,608,060	20.66	96,043,836	18.63	106,083,764	17.92
13	294,573,648	22.46	422,938,080	20.37	466,189,268	19.64
14	1,296,739,560	24.31	1,854,194,080	22.21	2,039,686,412	21.47
15	5,706,787,808	26.13	8,139,479,608	24.01	8,943,399,564	23.28
16	25,029,783,540	28.01	35,603,096,872	25.91	39,068,913,604	25.20
17	109,780,078,372	29.87	155,900,872,104	27.79	170,957,960,396	27.09

**Table A7:** Properties of SAWs on BCC lattice with 3 layers between the confining planes (*n* = 3, Type 1, 2).

BCC (*n* = 3, Type 1)			BCC (*n* = 3, Type 2)	
*N*	$c_{N}$	$\langle {\left|\omega^{N}\right|}^{2}\rangle$	$c_{N}$	$\langle {\left|\omega^{N}\right|}^{2}\rangle$
1	4	1.000	8	1.000
2	28	2.286	24	1.778
3	84	3.032	168	3.032
4	560	4.495	448	4.381
5	1512	5.854	3024	5.854
6	10,024	7.471	7776	7.671
7	26,016	9.276	52,032	9.276
8	172,144	11.01	131,392	11.49
9	437,216	13.21	874,432	13.21
10	2,888,704	15.04	2,184,192	15.76
11	7,242,304	17.59	14,484,608	17.59
12	47,788,288	19.52	35,913,728	20.46
13	118,793,664	22.37	237,587,328	22.37
14	783,007,232	24.39	585,931,008	25.53
15	1,934,717,312	27.53	3,869,434,624	27.53

**Table A8:** Properties of SAWs on the BCC lattice with 4 layers between the confining planes (*n* = 4, Type 1, 2).

BCC (*n* = 4, Type 1)			BCC (*n* = 4, Type 2)	
*N*	$c_{N}$	$\langle {\left|\omega^{N}\right|}^{2}\rangle$	$c_{N}$	$\langle {\left|\omega^{N}\right|}^{2}\rangle$
1	4	1.000	8	1.000
2	28	2.286	40	2.133
3	148	3.883	216	2.975
4	752	4.482	1100	4.422
5	3928	6.279	5516	5.502
6	19,240	7.061	27,436	7.195
7	96,800	9.129	135,308	8.501
8	471,652	10.12	662,208	10.41
9	2,324,620	12.42	3,234,984	11.92
10	11,266,332	13.59	15,695,400	14.02
11	54,928,996	16.10	76,139,448	15.71
12	264,967,864	17.44	367,445,292	17.98
13	1,283,256,176	20.12	1,773,482,796	19.83
14	6,167,881,032	21.62	8,526,698,460	22.26
15	29,733,461,768	24.46	41,001,069,836	24.27

**Table A9:** Properties of SAWs on the BCC lattice with five layers between the confining plates (*n* = 5, Type 1, 2, 3).

BCC (*n* = 5, Type 1)			BCC (*n* = 5, Type 2)		BCC (*n* = 5, Type 3)	
*N*	$c_{N}$	$\langle {\left|\omega^{N}\right|}^{2}\rangle$	$c_{N}$	$\langle {\left|\omega^{N}\right|}^{2}\rangle$	$c_{N}$	$\langle {\left|\omega^{N}\right|}^{2}\rangle$
1	4	1.000	8	1.000	8	1.000
2	28	2.286	40	2.133	56	2.286
3	148	3.883	280	3.438	264	2.939
4	1008	5.376	1292	4.425	1752	4.432
5	4696	6.361	8700	5.904	8008	5.369
6	31,208	7.957	38,956	7.001	52,600	7.015
7	140,576	8.985	258,124	8.614	235,096	8.172
8	923,408	10.70	1,137,676	9.908	1,534,264	9.947
9	4,088,104	11.93	7,467,996	11.65	6,759,784	11.31
10	26,673,936	13.75	32,589,060	13.14	43,920,344	13.20
11	116,790,808	15.20	212,627,204	15.000	191,672,792	14.76
12	758,669,728	17.14	921,579,828	16.68	1,241,447,848	16.75
13	3,296,625,336	18.78	5,987,539,924	18.65	5,381,829,176	18.49
14	21,347,913,984	20.82	25,824,254,724	20.50	34,775,532,088	20.58
15	92,254,133,376	22.64	167,265,106,124	22.57	150,021,945,496	22.48

**Table A10:** Properties of SAWS on FCC lattice with 3 layers between the confining planes (*n* = 3, Type 1, 2).

FCC (*n* = 3, Type 1)			FCC (*n* = 3, Type 2)	
*N*	$c_{N}$	$\langle {\left|\omega^{N}\right|}^{2}\rangle$	$c_{N}$	$\langle {\left|\omega^{N}\right|}^{2}\rangle$
1	8	1.000	12	1.000
2	72	2.222	100	1.920
3	608	3.355	796	2.990
4	4876	4.523	6292	4.183
5	38,332	5.769	49,020	5.499
6	297,468	7.121	377,996	6.926
7	2,287,380	8.580	2,893,932	8.452
8	17,471,516	10.14	22,030,220	10.07
9	132,758,268	11.80	166,942,556	11.79
10	1,004,552,340	13.55	1,260,417,828	13.59
11	7,575,290,444	15.39	9,487,397,172	15.47
12	56,963,463,220	17.30	71,232,793,460	17.43

**Table A11:** Properties of SAWS on FCC lattice with 4 layers between the confining plates (*n* = 4, Type 1, 2).

FCC (*n* = 4, Type 1)			FCC (*n* = 4, Type 2)	
*N*	$c_{N}$	$\langle {\left|\omega^{N}\right|}^{2}\rangle$	$c_{N}$	$\langle {\left|\omega^{N}\right|}^{2}\rangle$
1	8	1.000	12	1.000
2	72	2.222	116	2.069
3	672	3.607	1036	3.147
4	6092	4.934	9024	4.285
5	53,676	6.208	77,752	5.501
6	464,316	7.487	665,008	6.790
7	3,972,740	8.805	5,653,120	8.153
8	33,748,832	10.18	47,804,044	9.589
9	285,181,384	11.62	402,465,316	11.10
10	2,399,555,928	13.13	3,376,047,476	12.68
11	20,118,990,904	14.71	28,233,689,900	14.32
12	168,187,509,900	16.37	235,510,903,272	16.03

**Table A12:** Properties of SAWs on FCC lattice with 5 layers between the confining plates (*n* = 5, Type 1, 2, 3).

FCC (*n* = 5, Type 1)			FCC (*n* = 5, Type 2)		FCC (*n* = 5, Type 3)	
*N*	$c_{N}$	$\langle {\left|\omega^{N}\right|}^{2}\rangle$	$c_{N}$	$\langle {\left|\omega^{N}\right|}^{2}\rangle$	$c_{N}$	$\langle {\left|\omega^{N}\right|}^{2}\rangle$
1	8	1.000	12	1.000	12	1,000
2	72	2.222	116	2.069	132	2.182
3	672	3.607	1100	3.313	1276	3.245
4	6348	5.138	10,240	4.558	11,756	4.340
5	59,564	6.634	93,864	5.810	106,484	5.501
6	550,524	8.062	852,080	7.087	957,524	6.730
7	5,021,572	9.447	7,680,816	8.397	8,578,324	8.022
8	45,364,428	10.82	68,862,952	9.750	76,622,980	9.375
9	407,048,708	12.21	614,763,576	11.15	682,422,404	10.79
10	3,634,621,916	13.62	5,469,051,720	12.60	6,060,924,172	12.26
11	32,334,144,252	15.07	48,511,115,392	14.10	53,692,606,892	13.78
12	286,791,329,140	16.57	429,222,436,536	15.65	475,067,855,437	16.03

In the continuation, we present results from the statistical analysis based on the PDF and the folded variant for the SAW size as a function of lattice type, SAW length, point of origin (Type), and level of confinement for selected systems. Calculated statistical variables include the mean value, the most repeated one and for the folded variant the top point, *μ*_H_, corresponding to the half of the distribution when the representation changes from upslope to downslope, and the mean absolute deviation, *σ*.

**Table A13:** Statistical parameters of SAW size for the SC restricted lattice as a function of system characteristics (fixed: *N* = 16) for selected systems.

System	$\langle {\left|\omega^{16}\right|}^{2}\rangle$	Most Repeated Value (PDF)	Folded Distribution	
			*µ*_H_ (Top Point)	*σ* (68.3%)
*n* = 1, Type 1	51.99	50	50	±32
*n* = 2, Type 1	32.65	26	26	±22
*n* = 3, Type 2	27.97	26	26	±20
*n* = 4, Type 2	26.12	26	22	±18
*n* = 5, Type 1	28.01	14	24	±16
*n* = 5, Type 2	25.91	14	22	±16
*n* = 5, Type 3	25.20	14	20	±16

**Table A14:** Statistical parameters of SAW size for the SC restricted lattice as a function of SAW steps (fixed: *n* = 5, Type 3) for selected systems.

*N*	$\langle {\left|\omega^{N}\right|}^{2}\rangle$	Most Repeated Value (PDF)	Folded Distribution	
			*µ*_H_ (Top Point)	*σ* (68.3%)
13	19.64	14	17	±13
14	21.47	14	18	±14
15	23.28	17	19	±16
16	25.20	14	20	±16

**Table A15:** Statistical parameters of SAW size for the BCC restricted lattice as a function of system characteristics (fixed: *N* = 14) for selected systems.

System	$\langle {\left|\omega^{14}\right|}^{2}\rangle$	Most Repeated Value (PDF)	Folded Distribution	
			*µ*_H_ (Top Point)	*σ* (68.3%)
*n* = 2, Type 1	28.52	34	27	±19
*n* = 3, Type 2	25.53	34	23	±17
*n* = 4, Type 2	22.26	14	19	±15
*n* = 5, Type 1	20.82	22	19	±14
*n* = 5, Type 2	20.50	14	18	±14
*n* = 5, Type 3	20.58	7	18	±14

**Table A16:** Statistical parameters of SAW size for the BCC restricted lattice as a function of SAW steps (fixed: *n* = 5, Type 3) for selected systems.

*N*	$\langle {\left|\omega^{N}\right|}^{2}\rangle$	Most Repeated Value (PDF)	Folded Distribution	
			*µ*_H_ (Top Point)	*σ* (68.3%)
11	14.76	17	12	±9
12	16.75	7	14	±11
13	18.49	12	17	±14
14	20.58	7	18	±14

**Table A17:** Statistical parameters of SAW size for the FCC restricted lattice as a function of system characteristics (fixed: *N* = 11) for selected systems.

System	$\langle {\left|\omega^{11}\right|}^{2}\rangle$	Most Repeated Value (PDF)	Folded Distribution	
			*µ*_H_ (Top Point)	*σ* (68.3%)
*n* = 2, Type 1	17.95	13	16	±12
*n* = 3, Type 2	15.47	13	13	±11
*n* = 4, Type 2	14.32	13	13	±10
*n* = 5, Type 1	15.07	7	13	±10
*n* = 5, Type 2	14.10	7	12	±10
*n* = 5, Type 3	13.78	7	12	±10

**Table A18:** Statistical parameters of SAW size for the FCC restricted lattice as a function of SAW steps (fixed: *n* = 5, Type 3) for selected systems.

*N*	$\langle {\left|\omega^{N}\right|}^{2}\rangle$	Most Repeated Value (PDF)	Folded Distribution	
			*µ*_H_ (Top Point)	*σ* (68.3%)
8	9.375	7	7	±7
9	10.79	7	9	±8
10	12.26	7	10	±9
11	13.78	13	12	±10

## References

[1] Hollahan J.R., Wydeven T., Johnson C.C. (1974). Combination moisture resistant and antireflection plasma polymerized thin-films for optical coatings. Appl. Opt..

[2] Zhao H.X., Prieto L., Pez L.O., Zhou X.Z., Deng X., Cui J.X. (2019). Multistimuli responsive liquid-release in dynamic polymer coatings for controlling surface slipperiness and optical performance. Adv. Mater. Interfaces.

[3] Nickmans K., van der Heijden D.A.C., Schenning A. (2019). Photonic shape memory chiral nematic polymer coatings with changing surface topography and color. Adv. Opt. Mater..

[4] Pan H.L., Li Y.N., Wu Y.L., Liu P., Ong B.S., Zhu S.P., Xu G. (2007). Low-temperature, solution-processed, high-mobility polymer semiconductors for thin-film transistors. J. Am. Chem. Soc..

[5] Sun J., Gerberich W.W., Francis L.F. (2003). Electrical and optical properties of ceramic-polymer nanocomposite coatings. J. Polym. Sci. Part B Polym. Phys..

[6] Greenham N.C., Peng X.G., Alivisatos A.P. (1996). Charge separation and transport in conjugated-polymer/semiconductor-nanocrystal composites studied by photoluminescence quenching and photoconductivity. Phys. Rev. B.

[7] Wang X.C., Maeda K., Chen X.F., Takanabe K., Domen K., Hou Y.D., Fu X.Z., Antonietti M. (2009). Polymer semiconductors for artificial photosynthesis: Hydrogen evolution by mesoporous graphitic carbon nitride with visible light. J. Am. Chem. Soc..

[8] Moller S., Perlov C., Jackson W., Taussig C., Forrest S.R. (2003). A polymer/semiconductor write-once read-many-times memory. Nature.

[9] Ji J.J., Wu X.M., Deng P., Zhou D.G., Lai D.X., Zhan H.B., Chen H.P. (2019). Impact of new skeletal isomerization in polymer semiconductors. J. Mater. Chem. C.

[10] Nomura K., Ohta H., Takagi A., Kamiya T., Hirano M., Hosono H. (2004). Room-temperature fabrication of transparent flexible thin-film transistors using amorphous oxide semiconductors. Nature.

[11] Fortunato E.M.C., Barquinha P.M.C., Pimentel A., Goncalves A.M.F., Marques A.J.S., Pereira L.M.N., Martins R.F.P. (2005). Fully transparent zno thin-film transistor produced at room temperature. Adv. Mater..

[12] Kim M.G., Kanatzidis M.G., Facchetti A., Marks T.J. (2011). Low-temperature fabrication of high-performance metal oxide thin-film electronics via combustion processing. Nat. Mater..

[13] Wang J.T., Li P., Zhang Y.F., Liu Y.R., Wu W.J., Liu J.D. (2019). Porous nafion nanofiber composite membrane with vertical pathways for efficient through-plane proton conduction. J. Membr. Sci..

[14] Hwang M., Karenson M.O., Elabd Y.A. (2019). High production rate of high purity, high fidelity nafion nanofibers via needleless electrospinning. ACS Appl. Polym. Mater..

[15] Hoare T.R., Kohane D.S. (2008). Hydrogels in drug delivery: Progress and challenges. Polymer.

[16] Calo E., Khutoryanskiy V.V. (2015). Biomedical applications of hydrogels: A review of patents and commercial products. Eur. Polym. J..

[17] Czakkel O., Berke B., Laszlo K. (2019). Effect of graphene-derivatives on the responsivity of pnipam-based thermosensitive nanocomposites—A review. Eur. Polym. J..

[18] Zelikin A.N. (2010). Drug releasing polymer thin films: New era of surface-mediated drug delivery. ACS Nano.

[19] Chen X.C., Huang W.P., Hu M., Ren K.F., Ji J. (2019). Controlling structural transformation of polyelectrolyte films for spatially encapsulating functional species. Small.

[20] Alder B.J., Wainwright T.E. (1957). Phase transition for a hard sphere system. J. Chem. Phys..

[21] Auer S., Frenkel D. (2001). Prediction of absolute crystal-nucleation rate in hard-sphere colloids. Nature.

[22] Auer S., Frenkel D. (2004). Quantitative prediction of crystal-nucleation rates for spherical colloids: A computational approach. Annu. Rev. Phys. Chem..

[23] Cheng Z.D., Chaikin P.M., Zhu J.X., Russel W.B., Meyer W.V. (2002). Crystallization kinetics of hard spheres in microgravity in the coexistence regime: Interactions between growing crystallites. Phys. Rev. Lett..

[24] Cheng Z.D., Russell W.B., Chaikin P.M. (1999). Controlled growth of hard-sphere colloidal crystals. Nature.

[25] De Villeneuve V.W.A., Dullens R.P.A., Aarts D., Groeneveld E., Scherff J.H., Kegel W.K., Lekkerkerker H.N.W. (2005). Colloidal hard-sphere crystal growth frustrated by large spherical impurities. Science.

[26] Dolbnya I.P., Petukhov A.V., Aarts D., Vroege G.J., Lekkerkerker H.N.W. (2005). Coexistence of rhcp and fcc phases in hard-sphere colloidal crystals. Europhys. Lett..

[27] Gast A.P., Monovoukas Y. (1991). A new growth instability in colloidal crystallization. Nature.

[28] Harland J.L., vanMegen W. (1997). Crystallization kinetics of suspensions of hard colloidal spheres. Phys. Rev. E.

[29] Henderson S.I., van Megen W. (1998). Metastability and crystallization in suspensions of mixtures of hard spheres. Phys. Rev. Lett..

[30] Iacopini S., Palberg T., Schope H.J. (2009). Ripening-dominated crystallization in polydisperse hard-sphere-like colloids. Phys. Rev. E.

[31] Iacopini S., Palberg T., Schope H.J. (2009). Crystallization kinetics of polydisperse hard-sphere-like microgel colloids: Ripening dominated crystal growth above melting. J. Chem. Phys..

[32] Martin S., Bryant G., van Megen W. (2005). Crystallization kinetics of polydisperse colloidal hard spheres. Ii. Binary mixtures. Phys. Rev. E.

[33] O’Malley B., Snook I. (2003). Crystal nucleation in the hard sphere system. Phys. Rev. Lett..

[34] O’Malley B., Snook I. (2005). Structure of hard-sphere fluid and precursor structures to crystallization. J. Chem. Phys..

[35] Punnathanam S., Monson P.A. (2006). Crystal nucleation in binary hard sphere mixtures: A monte carlo simulation study. J. Chem. Phys..

[36] Pusey P.N., Vanmegen W. (1986). Phase-behavior of concentrated suspensions of nearly hard colloidal spheres. Nature.

[37] Pusey P.N., Vanmegen W., Bartlett P., Ackerson B.J., Rarity J.G., Underwood S.M. (1989). Structure of crystals of hard colloidal spheres. Phys. Rev. Lett..

[38] Rintoul M.D., Torquato S. (1996). Metastability and crystallization in hard-sphere systems. Phys. Rev. Lett..

[39] Schilling T., Schope H.J., Oettel M., Opletal G., Snook I. (2010). Precursor-mediated crystallization process in suspensions of hard spheres. Phys. Rev. Lett..

[40] Toth G.I., Granasy L. (2009). Crystal nucleation in the hard-sphere system revisited: A critical test of theoretical approaches. J. Phys. Chem. B.

[41] Zaccarelli E., Valeriani C., Sanz E., Poon W.C.K., Cates M.E., Pusey P.N. (2009). Crystallization of hard-sphere glasses. Phys. Rev. Lett..

[42] Zhu J.X., Li M., Rogers R., Meyer W., Ottewill R.H., Russell W.B., Chaikin P.M. (1997). Crystallization of hard-sphere colloids in microgravity. Nature.

[43] Karayiannis N.C., Foteinopoulou K., Laso M. (2009). Entropy-driven crystallization in dense systems of athermal chain molecules. Phys. Rev. Lett..

[44] Karayiannis N.C., Foteinopoulou K., Abrams C.F., Laso M. (2010). Modeling of crystal nucleation and growth in athermal polymers: Self-assembly of layered nano-morphologies. Soft Matter.

[45] Karayiannis N.C., Foteinopoulou K., Laso M. (2013). Spontaneous crystallization in athermal polymer packings. Int. J. Mol. Sci..

[46] Karayiannis N.C., Foteinopoulou K., Laso M. (2013). Jamming and crystallization in athermal polymer packings. Philos. Mag..

[47] Karayiannis N.C., Laso M. (2008). Dense and nearly jammed random packings of freely jointed chains of tangent hard spheres. Phys. Rev. Lett..

[48] Karayiannis N.C., Foteinopoulou K., Laso M. (2009). The structure of random packings of freely jointed chains of tangent hard spheres. J. Chem. Phys..

[49] Laso M., Karayiannis N.C., Foteinopoulou K., Mansfield M.L., Kroger M. (2009). Random packing of model polymers: Local structure, topological hindrance and universal scaling. Soft Matter.

[50] Hoy R.S. (2017). Jamming of semiflexible polymers. Phys. Rev. Lett..

[51] Ni R., Dijkstra M. (2013). Effect of bond length fluctuations on crystal nucleation of hard bead chains. Soft Matter.

[52] Karayiannis N.C., Foteinopoulou K., Laso M. (2015). The role of bond tangency and bond gap in hard sphere crystallization of chains. Soft Matter.

[53] Shakirov T., Paul W. (2018). Crystallization in melts of short, semiflexible hard polymer chains: An interplay of entropies and dimensions. Phys. Rev. E.

[54] Shakirov T. (2019). Crystallisation in melts of short, semi-flexible hard-sphere polymer chains: The role of the non-bonded interaction range. Entropy.

[55] Karayiannis N.C., Laso M. (2008). Monte carlo scheme for generation and relaxation of dense and nearly jammed random structures of freely jointed hard-sphere chains. Macromolecules.

[56] Pant P.V.K., Theodorou D.N. (1995). Variable connectivity method for the atomistic monte-carlo simulation of polydisperse polymer melts. Macromolecules.

[57] Mavrantzas V.G., Boone T.D., Zervopoulou E., Theodorou D.N. (1999). End-bridging monte carlo: A fast algorithm for atomistic simulation of condensed phases of long polymer chains. Macromolecules.

[58] Karayiannis N.C., Giannousaki A.E., Mavrantzas V.G., Theodorou D.N. (2002). Atomistic monte carlo simulation of strictly monodisperse long polyethylene melts through a generalized chain bridging algorithm. J. Chem. Phys..

[59] Karayiannis N.C., Mavrantzas V.G., Theodorou D.N. (2002). A novel monte carlo scheme for the rapid equilibration of atomistic model polymer systems of precisely defined molecular architecture. Phys. Rev. Lett..

[60] Karayiannis N.C., Foteinopoulou K., Laso M. (2009). Contact network in nearly jammed disordered packings of hard-sphere chains. Phys. Rev. E.

[61] Laso M., Karayiannis N.C. (2008). Flexible chain molecules in the marginal and concentrated regimes: Universal static scaling laws and cross-over predictions. J. Chem. Phys..

[62] Foteinopoulou K., Karayiannis N.C., Laso M., Kroger M. (2009). Structure, dimensions, and entanglement statistics of long linear polyethylene chains. J. Phys. Chem. B.

[63] Ramos P.M., Karayiannis N.C., Laso M. (2018). Off-lattice simulation algorithms for athermal chain molecules under extreme confinement. J. Comput. Phys..

[64] Benito J., Karayiannis N.C., Laso M. (2018). Confined polymers as self-avoiding random walks on restricted lattices. Polymers.

[65] Bicout D.J., Kats E.I., Petukhov A.K., Whitney R.S. (2013). Size independence of statistics for boundary collisions of random walks and its implications for spin-polarized gases. Phys. Rev. Lett..

[66] Weiss G.H., Rubin R.J. (1983). Random-walks—Theory and selected applications. Adv. Chem. Phys..

[67] Chew W.X., Kaizu K., Watabe M., Muniandy S.V., Takahashi K., Arjunan S.N.V. (2019). Surface reaction-diffusion kinetics on lattice at the microscopic scale. Phys. Rev. E.

[68] Brydges D., Frohlich J., Spencer T. (1982). The random-walk representation of classical spin systems and correlation inequalities. Commun. Math. Phys..

[69] Granzotti C.R.F., Ribeiro F.L., Martinez A.S., da Silva M.A.A. (2019). Persistence length convergence and universality for the self-avoiding random walk. J. Phys. Math. Theor..

[70] Scalas E. (2006). The application of continuous-time random walks in finance and economics. Phys. Stat. Mech. Appl..

[71] Martinez I.A., Bisker G., Horowitz J.M., Parrondo J.M.R. (2019). Inferring broken detailed balance in the absence of observable currents. Nat. Commun..

[72] Zhao J.Z., Wang P.H., Lui J.C.S., Towsley D., Guan X.H. (2019). Sampling online social networks by random walk with indirect jumps. Data Min. Knowl. Discov..

[73] Gkantsidis C., Mihail M., Saberi A. (2006). Random walks in peer-to-peer networks: Algorithms and evaluation. Perform. Eval..

[74] Zhao J.Z., Wang P.H., Lui J.C.S. (2019). Optimizing node discovery on networks: Problem definitions, fast algorithms, and observations. Inf. Sci..

[75] Boyer D., Solis-Salas C. (2014). Random walks with preferential relocations to places visited in the past and their application to biology. Phys. Rev. Lett..

[76] Codling E.A., Plank M.J., Benhamou S. (2008). Random walk models in biology. J. R. Soc. Interface.

[77] Evans D.R., Kwak H.S., Giesen D.J., Goldberg A., Halls M.D., Oh-e M. (2016). Estimation of charge carrier mobility in amorphous organic materials using percolation corrected random-walk model. Org. Electron..

[78] Khan M., Mason T.G. (2014). Random walks of colloidal probes in viscoelastic materials. Phys. Rev. E.

[79] Limoge Y., Bocquet J.L. (1990). Temperature behavior of tracer diffusion in amorphous materials—A random-walk approach. Phys. Rev. Lett..

[80] Karayiannis N.C., Mavrantzas V.G., Theodorou D.N. (2001). Diffusion of small molecules in disordered media: Study of the effect of kinetic and spatial heterogeneities. Chem. Eng. Sci..

[81] Apostolopoulou M., Dusterhoft R., Day R., Stamatakis M., Coppens M.O., Striolo A. (2019). Estimating permeability in shales and other heterogeneous porous media: Deterministic vs. Stochastic investigations. Int. J. Coal Geol..

[82] Grady L. (2006). Random walks for image segmentation. IEEE Trans. Pattern Anal. Mach. Intell..

[83] Shen J.B., Du Y.F., Wang W.G., Li X.L. (2014). Lazy random walks for superpixel segmentation. IEEE Trans. Image Process..

[84] Shen R., Cheng I., Shi J.B., Basu A. (2011). Generalized random walks for fusion of multi-exposure images. IEEE Trans. Image Process..

[85] Kesten H. (1963). On number of self-avoiding walks. J. Math. Phys..

[86] Hammond A. (2019). On self-avoiding polygons and walks: The snake method via pattern fluctuation. Trans. Am. Math. Soc..

[87] Fisher M.E., Sykes M.F. (1959). Excluded-volume problem and the ising model of ferromagnetism. Phys. Rev..

[88] Janse van Rensburg E.J. (2015). The Statistical Mechanics of Interacting Walks, Polygons, Animals and Vesicles.

[89] Risken H. (1996). The Fokker-Planck Equation: Methods of Solution and Applications.

[90] Gardiner C. (2009). Stochastic Methods: A Handbook for the Natural and Social Sciences.

[91] Hutchcroft T. (2019). Self-avoiding walk on nonunimodular transitive graphs. Ann. Probab..

[92] deGennes P.G. (1980). Scaling Concepts in Polymer Physics.

[93] Rubinstein M., Colby R.H. (2003). Polymer Physics (Chemistry).

[94] Weiss G.H. (1994). Aspects and Applications of the Random Walk.

[95] Stauffer D., Aharony A. (2014). Introduction to Percolation Theory.

[96] Ottinger H.C. (2012). Stochastic Processes in Polymeric Fluids.

[97] Blavatska V., Janke W. (2009). Walking on fractals: Diffusion and self-avoiding walks on percolation clusters. J. Phys. Math. Theor..

[98] Dagrosa E., Owczarek A.L., Prellberg T. (2017). Writhe induced phase transition in unknotted self-avoiding polygons. J. Stat. Mech. Theory Exp..

[99] Rubin R.J. (1952). The excluded volume effect in polymer chains and the analogous random walk problem. J. Chem. Phys..

[100] Dewey T.G. (1999). Statistical mechanics of protein sequences. Phys. Rev. E.

[101] Rubin R.J. (1965). Random-walk model of chain-polymer adsorption at a surface. J. Chem. Phys..

[102] Yang Q.H., Yang X., Luo M.B. (2019). Adsorption of polymer chains on heterogeneous surfaces with random adsorption sites. Polymer.

[103] Wall F.T., Erpenbeck J.J. (1959). New method for the statistical computation of polymer dimensions. J. Chem. Phys..

[104] Fisher M.E. (1966). Shape of a self-avoiding walk or polymer chain. J. Chem. Phys..

[105] Helfand E. (1975). Theory of inhomogeneous polymers—Fundamentals of gaussian random-walk model. J. Chem. Phys..

[106] Dimarzio E.A. (1991). Statistics of a polymer molecule in the presence of asymmetric obstacles. Macromolecules.

[107] James E.W., Soteros C.E., Whittington S.G. (2003). Localization of a random copolymer at an interface: An exact enumeration study. J. Phys. Math. Gen..

[108] Alvarez J., van Rensburg E.J.J., Soteros C.E., Whittington S.G. (2008). Self-avoiding polygons and walks in slits. J. Phys. Math. Theor..

[109] Whittington S.G., Soteros C.E. (1992). Uniform branched polymers in confined geometries. J. Macromol. Sci. Pure Appl. Chem..

[110] Bradly C.J., van Rensburg E.J.J., Owczarek A.L., Whittington S.G. (2019). Force-induced desorption of 3-star polymers in two dimensions. J. Phys. Math. Theor..

[111] Beaton N.R., Eng J.W., Soteros C.E. (2019). Knotting statistics for polygons in lattice tubes. J. Phys. Math. Theor..

[112] Orr W.J.C. (1947). Statistical treatment of polymer solutions at infinite dilution. Trans. Faraday Soc..

[113] Jaleel A.A.A., Ponmurugan M., Rajesh R., Satyanarayana S.V.M. (2018). Phase transitions in a linear self-interacting polymer on fcc lattice using flat energy interacting growth walk algorithm. J. Stat. Mech. Theory Exp..

[114] Zivic I., Elezovic-Hadzic S., Milosevic S. (2018). Semiflexible polymer chains on the square lattice: Numerical study of critical exponents. Phys. Rev. E.

[115] Marcetic D., Elezovic-Hadzic S., Adzic N., Zivic I. (2019). Semi-flexible compact polymers in two dimensional nonhomogeneous confinement. J. Phys. Math. Theor..

[116] Edwards S.F., Freed K.F. (1969). Entropy of a confined polymer I. J. Phys. Part Gen..

[117] Mishra P.K. (2015). Equilibrium statistics of an infinitely long chain in the severe confined geometry: Exact results. Phase Transit..

[118] Mishra P.K. (2017). Effect of confinement and stiffness on the conformational change of a semiflexible homopolymer chain. Indian J. Phys..

[119] Brak R., Iliev G.K., Owczarek A.L., Whittington S.G. (2010). The exact solution of a three-dimensional lattice polymer confined in a slab with sticky walls. J. Phys. Math. Theor..

[120] Soteros C.E., DeGier J., Warnaar O. (2006). Eulerian graph embeddings and trails confined to lattice tubes. International Workshop on Statistical Mechanics and Combinatorics: Counting Complexity.

[121] Wall F.T., Seitz W.A., Chin J.C., Degennes P.G. (1978). Statistics of self-avoiding walks confined to strips and capillaries. Proc. Natl. Acad. Sci. USA.

[122] Sykes M.F. (1963). Self-avoiding walks on simple cubic lattice. J. Chem. Phys..

[123] Guttmann A.J. (1989). On the critical-behavior of self-avoiding walks. 2. J. Phys. Math. Gen..

[124] Clisby N. (2017). Scale-free monte carlo method for calculating the critical exponent. Of self-avoiding walks. J. Phys. Math. Theor..

[125] MacDonald D., Joseph S., Hunter D.L., Moseley L.L., Jan N., Guttmann A.J. (2000). Self-avoiding walks on the simple cubic lattice. J. Phys. Math. Gen..

[126] Conway A.R., Enting I.G., Guttmann A.J. (1993). Algebraic techniques for enumerating self-avoiding walks on the square lattice. J. Phys. Math. Gen..

[127] Conway A.R., Guttmann A.J. (1996). Square lattice self-avoiding walks and corrections to scaling. Phys. Rev. Lett..

[128] Macdonald D., Hunter D.L., Kelly K., Jan N. (1992). Self-avoiding walks in 2 to 5 dimensions—Exact enumerations and series study. J. Phys. Math. Gen..

[129] Clisby N. (2010). Efficient implementation of the pivot algorithm for self-avoiding walks. J. Stat. Phys..

[130] Clisby N. (2018). Monte carlo study of four-dimensional self-avoiding walks of up to one billion steps. J. Stat. Phys..

[131] Schram R.D., Barkema G.T., Bisseling R.H. (2011). Exact enumeration of self-avoiding walks. J. Stat. Mech. Theory Exp..

[132] Schram R.D., Barkema G.T., Bisseling R.H. (2013). Sawdoubler: A program for counting self-avoiding walks. Comput. Phys. Commun..

[133] Schram R.D., Barkema G.T., Bisseling R.H., Clisby N. (2017). Exact enumeration of self-avoiding walks on bcc and fcc lattices. J. Stat. Mech. Theory Exp..

[134] Humphrey W., Dalke A., Schulten K. (1996). Vmd: Visual molecular dynamics. J. Mol. Graph. Model..

[135] Karayiannis N.C., Foteinopoulou K., Laso M. (2009). The characteristic crystallographic element norm: A descriptor of local structure in atomistic and particulate systems. J. Chem. Phys..

[136] Karayiannis N.C., Malshe R., Kroger M., de Pablo J.J., Laso M. (2012). Evolution of fivefold local symmetry during crystal nucleation and growth in dense hard-sphere packings. Soft Matter.

[137] Jensen I. (2004). Self-avoiding walks and polygons on the triangular lattice. J. Stat. Mech. Theory Exp..

[138] Clisby N., Dunweg B. (2016). High-precision estimate of the hydrodynamic radius for self-avoiding walks. Phys. Rev. E.

[139] Clisby N. (2010). Accurate estimate of the critical exponent nu for self-avoiding walks via a fast implementation of the pivot algorithm. Phys. Rev. Lett..

[140] Duminil-Copin H., Smirnov S. (2012). The connective constant of the honeycomb lattice equals root 2+root 2. Ann. Math..

[141] Nienhuis B. (1984). Critical-behavior of two-dimensional spin models and charge asymmetry in the coulomb gas. J. Stat. Phys..

[142] Nienhuis B. (1982). Exact critical-point and critical exponents of o(n) models in 2 dimensions. Phys. Rev. Lett..

