# Supplementary material for: Self-Avoiding Random Walks as a Model to Study Athermal Linear Polymers under Extreme Plate Confinement
Source: Polymers (Basel). 2020 Apr 3;12(4):799. doi: 10.3390/polym12040799 (PMC7240602; doi:10.3390/polym12040799)
Supplement: Supplementary file 1 [file polymers-12-00799-s001.zip › Figure03a_3D.pdf]

This area requires a 3D PDF enabled viewer such as Adobe Reader.
